# A Rare Case of Left-Sided Gastroschisis in a Human Museum Specimen

**DOI:** 10.7759/cureus.28995

**Published:** 2022-09-09

**Authors:** Gyanaranjan Nayak, Niranjan Sahoo, Sujita Pradhan, Gyanraj Singh, Sitansu K Panda

**Affiliations:** 1 Anatomy, Institute of Medical Sciences (IMS) and SUM Hospital, Siksha 'O' Anusandhan Deemed to be University, Bhubaneswar, IND; 2 Forensic Medicine, All India Institute of Medical Sciences (AIIMS) Bhopal, Bhopal, IND; 3 Anatomy, Kalinga Institute of Medical Sciences (KIMS) Kalinga Institute of Industrial Technology (KIIT) Deemed to be University, Bhubaneswar, IND

**Keywords:** stillbirth, intrauterine growth retardation, premature birth, congenital anomaly, anterior abdominal wall defect, gastroschisis

## Abstract

Gastroschisis is a congenital defect in the anterior abdominal wall resulting in herniation of the abdominal viscera without any fetal membrane covering it. It usually occurs to the right of a normally inserted umbilical cord. The anomaly is associated with intrauterine growth retardation, stillbirth, and preterm delivery. We found a preserved specimen of a 17- to 20-week-old male human fetus presenting with gastroschisis in the Departmental Museum of Anatomy of the Institute of Medical Sciences and SUM Hospital, Bhubaneshwar, a medical college in Eastern India. The fetus showed a hiatus on the left side in the infraumbilical portion of the anterior abdominal wall with evisceration of the liver, spleen, coils of the small intestine, and a segment of the large intestine. The fetus otherwise had no obvious gross abnormality. The case is of particular interest as the incidence of left-sided gastroschisis is very rare.

## Introduction

Gastroschisis is a congenital defect in the anterior abdominal wall leading to the evisceration of abdominal viscera such as the small intestine, stomach, colon, or gonads. The defect is usually right-sided. The protruding viscera are devoid of any covering and thus exposed to amniotic fluid and the external environment leading to injuries and infection [1,2].

The incidence of gastroschisis has increased in recent times with a worldwide incidence of 1:3,000 to 1:10,000 births. Gastroschisis has a good prognosis and patient survival if cases are adequately managed [3,4]. However, there is an augmented risk of intrauterine growth retardation, premature delivery, and fetal loss associated with gastroschisis, particularly in middle- to low-income countries. In middle- to low-income countries, the associated factors with high mortality are the absence of a prenatal diagnosis, prematurity, delayed surgery, parenteral nutrition, mechanical ventilation, lack of intensive care facilities, and delivery outside tertiary care centers [5]. In high-income countries, factors associated with increased mortality are low birth weight, prematurity, and the presence of complications such as bowel atresia, necrotizing enterocolitis, and other congenital malformations [5]. The associated malformations (incidence: 5%-20%) include amyoplasia, limb anomalies, renal defects, and cardiac abnormalities [6].

Gastroschisis is usually detected during serial antenatal ultrasonography [7]. Due to the increased mortality of affected fetuses, particularly in low socioeconomic status countries, it is imperative to focus on such cases. We have attempted to highlight one such case in the current case report.

## Case presentation

**Figure 1:**
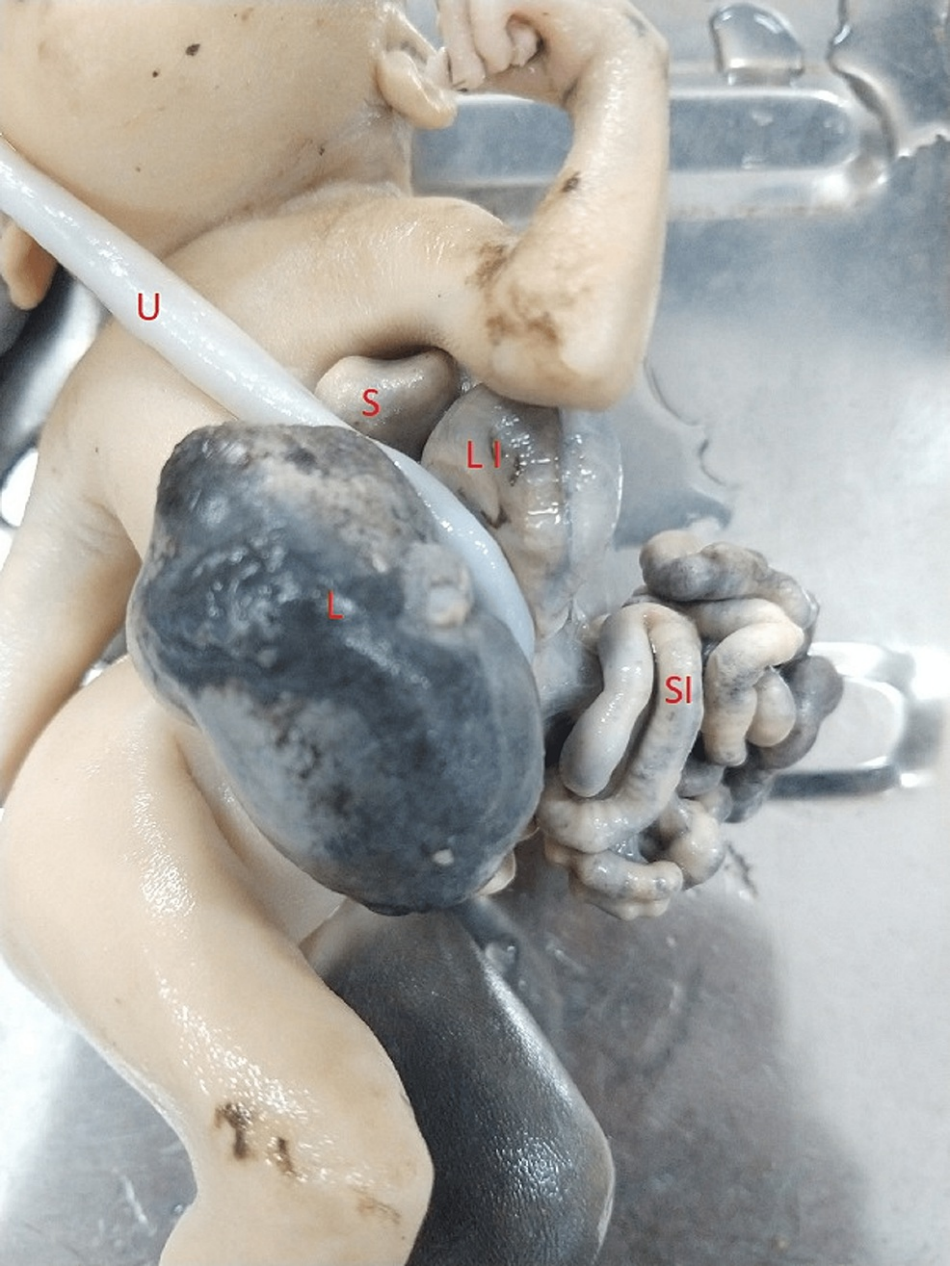
Fetus with gastroschisis showing eviscerations. The umbilical cord (U) is intact. L: liver; S: spleen; SI: small intestine; LI: large intestine

We have studied the museum specimen of a 17- to 20-week-old male fetus presenting with gastroschisis (Figure 1). The case in point showed left-sided gastroschisis in the infraumbilical location. The fetus showed features conducive to a gestational age of 17-20 weeks. The crown-rump length was 17.5 cm. The head circumference was 16 cm. The fetus showed complete limbs. The external genitalia was complete as well, with a well-defined scrotum and penis. However, the testes were undescended. Auricles and eyes could be distinguished. The fetus showed the presence of eyebrows and scalp hair. The placenta and umbilical cords were intact. There was a defect in the left infraumbilical region of the anterior abdominal wall showing the evisceration of various intra-abdominal organs (Figure 1). The defect was to the left of the normal umbilical ring. The umbilical cord was, as usual, attached to the umbilicus of the fetus. The viscera protruding through the defect included the liver, spleen, coils of the small intestine, and a segment of the large intestine. The fetus was otherwise normal. The dimensions of the infraumbilical defect could not be measured due to the host of viscera protruding through it.

## Discussion

Gastroschisis has been reported by various authors [7-10]. However, the cases reported belonged to live fetuses either in utero (by antenatal ultrasonography) or just after delivery. We have described gastroschisis in a human museum specimen.

The anterior abdominal wall defect in gastroschisis typically manifests on the right side. However, we have reported left-sided gastroschisis. Patel et al. [10] have reported one such case, and they have discussed 13 other such cases in which the gastroschisis is to the left of the umbilical ring.

Gastroschisis should be differentiated from omphalocele. Omphalocele is the herniation of abdominal viscera through the enlarged umbilical ring, and the protruding viscera have a covering of amnion [7]. On the other hand, gastroschisis is the protrusion of abdominal viscera usually through an opening to the right of the umbilical ring without any fetal membrane covering it.

There is a possible embryological basis for gastroschisis [11,12]. Gastroschisis is caused either by an occluded omphalomesenteric artery or early atrophy of the right umbilical vein before the fourth week of gestation. This results in infarction and subsequent rupture of the anterior abdominal wall, which causes protrusion of the abdominal viscera through the defect. Another theory [13,14] advocates that defective inclusion of the yolk sac in the fetal body stem leads to an additional opening in the anterior abdominal wall, and the viscera protrude outside the abdominal cavity.

Gastroschisis is usually detected during antenatal ultrasonography of expecting mothers around 18-20 weeks of gestation [2]. The associated risk factors are Caucasian race, Hispanic mother, young primigravida (<20 years), maternal malnutrition, prematurity of the fetus, low birth weight, exposure to nitrosamines, exposure to teratogens and agrochemicals during pregnancy, consumption of nonsteroidal anti-inflammatory drugs in the first trimester, smoking and alcohol, and illicit drug abuse [1,2,4,13,14].

Once the prenatal diagnosis of gastroschisis is made, a multidisciplinary approach is required for its optimal management that involves obstetricians, pediatric surgeons, and neonatologists [15].

Some studies report that there are no differences in the outcome of neonates diagnosed prenatally [16]. Such studies are made in developed countries where the delivery of the baby is done in centers with state-of-the-art facilities. However, it is important to recognize gastroschisis early in developing countries so that the cases can be referred timely to higher centers for their effective management.

Several studies put forward that early cesarean section decreases morbidity with respect to vaginal delivery. This can be attributed to the fact that babies born by vaginal delivery have a greater chance of infection of the protruding viscera or their perforation [16]. However, some authors do not report any such significant differences [15,17].

## Conclusions

Gastroschisis has a low incidence and good prognosis if properly managed. It requires adequate competency not only from the specialists but also from the primary care health personnel as appropriate and timely referral needs to be done to higher centers to avoid complications. Our study describes left-sided gastroschisis, which is very rare. We have described the case of gastroschisis in a museum specimen of a fetus approximately 17-20 weeks of age. Therefore, this study puts forward that timely diagnosis, referral, and management are essential in such cases to prevent fetal mortality and prematurity. We have noted that many viscera such as the liver, spleen, small intestine, and large intestine can protrude through the defect in gastroschisis. Thus, we advocate cesarean section to be the mode of delivery in such cases to prevent injury or perforation of the protruding viscera.

## References

[1] Islam S (2012). Advances in surgery for abdominal wall defects: gastroschisis and omphalocele. Clin Perinatol.

[2] Frolov P, Alali J, Klein MD (2010). Clinical risk factors for gastroschisis and omphalocele in humans: a review of the literature. Pediatr Surg Int.

[3] Waller SA, Paul K, Peterson SE, Hitti JE (2010). Agricultural-related chemical exposures, season of conception, and risk of gastroschisis in Washington State. Am J Obstet Gynecol.

[4] Torres US, Portela-Oliveira E, Braga Fdel C, Werner H Jr, Daltro PA, Souza AS (2015). When closure fails: what the radiologist needs to know about the embryology, anatomy, and prenatal imaging of ventral body wall defects. Semin Ultrasound CT MR.

[5] Marshall Niles SG, Mitchell-Fearon K, Gill MI (2017). Mortality-related factors in gastroschisis - a Jamaican perspective. J Pediatr Surg.

[6] Bergholz R, Boettcher M, Reinshagen K, Wenke K (2014). Complex gastroschisis is a different entity to simple gastroschisis affecting morbidity and mortality-a systematic review and meta-analysis. J Pediatr Surg.

[7] Joshi S, Singhavi S, Dedhia K (2017). A case report: gastroschisis. J Matern-Fetal Neonatal Med.

[8] Ibara-Calderon R, Gutiérrez-Montufar OO, Savedra-Torres JS, Zuniga Ceron LF (2018). Gastroschisis. Case report and management in primary care services. Case Rep.

[9] Morazán AF, Andrade DS, Torres SJ, Zelaya WP, Izaguirre RE, Molina FA, Gonzalez CH (2017). Non-viable neonatal gastroschisis: case report. J Clin Epigenet.

[10] Patel RV, More B, Sinha CK, Rajimawale A (2013). Inferior gastroschisis. BMJ Case Rep.

[11] Jones KL, Benirschke K, Chambers CD (2009). Gastroschisis: etiology and developmental pathogenesis. Clin Genet.

[12] Stevenson RE, Rogers RC, Chandler JC, Gauderer MW, Hunter AG (2009). Escape of the yolk sac: a hypothesis to explain the embryogenesis of gastroschisis. Clin Genet.

[13] Tassin M, Benachi A (2014). Diagnosis of abdominal wall defects in the first trimester. Curr Opin Obstet Gynecol.

[14] Poddar R, Hartley L (2009). Exomphalos and gastroschisis. Cont Educ Anaes Crit Care Pain.

[15] Gamba P, Midrio P (2014). Abdominal wall defects: prenatal diagnosis, newborn management, and long-term outcomes. Semin Pediatr Surg.

[16] Murphy FL, Mazlan TA, Tarheen F, Corbally MT, Puri P (2007). Gastroschisis and exomphalos in Ireland 1998-2004. Does antenatal diagnosis impact on outcome?. Pediatr Surg Int.

[17] Christison-Lagay ER, Kelleher CM, Langer JC (2011). Neonatal abdominal wall defects. Semin Fetal Neonatal Med.

